# Effectiveness of Indonesian house dust mite allergenic extract in triggering allergic rhinitis sensitivity in a mouse model: A preliminary study

**DOI:** 10.14202/vetworld.2022.2333-2341

**Published:** 2022-09-29

**Authors:** Yusuf Alif Pratama, Fakhriyah Dinina, Ahmad Dzulfikri Nurhan, Winda Fatma Sari, Chrismawan Ardianto, Junaidi Khotib

**Affiliations:** 1 Master Program of Pharmaceutical Science, Faculty of Pharmacy, Universitas Airlangga, Surabaya 60115, Indonesia; 2 Bachelor Program of Pharmacy, Faculty of Pharmacy, Universitas Airlangga, Surabaya 60115, Indonesia; 3 Department of Pharmacy Practice, Faculty of Pharmacy, Universitas Airlangga, Surabaya 60115, Indonesia

**Keywords:** allergen immunotherapy, allergic healing, allergic rhinitis, Indonesian house dust mites, neglected disease

## Abstract

**Background and Aim:**

Perennial allergic rhinitis (AR) is a chronic upper respiratory disease, with inflammation mediated by immunoglobulin E in the nasal mucosa caused by house dust mites. Recently, allergen immunotherapy showed promising allergic healing in patients with a definite history of sensitization. Based on this finding, a product was developed using Indonesian house dust mite (IHDM). This study aimed to optimize the allergenic rhinitis mouse model that was generated using IHDM to test the *in vivo* sensitivity and safety of this product.

**Materials and Methods:**

Seven groups of mice were used for effectiveness testing – normal, negative control with IHDM challenge, positive control with 0.1% histamine challenge, and AR group by both IHDM-induced sensitization at 12.5, 50, 250, or 500 μg and IHDM challenge. Mice were sensitized by intraperitoneal administration of IHDM once a week for 3 consecutive weeks. Thereafter, the challenge was given intranasally 5 times on alternate days. The number of nose rubbing and sneezing was noted. Eosinophil infiltration was assessed histologically using hematoxylin and eosin staining. The expression of interleukin-5 (IL-5) mRNA in the nasal mucosa was determined using semi-quantitative reverse transcription-polymerase chain reaction.

**Results:**

The induction of AR with IHDM significantly increased the number of nose rubbing and sneezing in the mouse model. Eosinophil infiltration was observed in the nasal mucosa; however, no significant change occurred in the expression of IL-5 mRNA.

**Conclusion:**

Overall, these data indicate that IHDM allergenic extract could be an effective sensitizing agent in a mouse model of AR. Although the use of IHDM is a limitation of this study because other sources of house dust mites might have different effects, this study provides a proper model for immunotherapy effectivity testing for *in vivo* pre-clinical studies.

## Introduction

Allergic rhinitis (AR) is a major public respiratory problem affecting approximately 40% of the worldwide population [1, 2]. In Indonesia, the prevalence of AR is 5%–45% of its population, which matches with the prevalence rate in Asia [3]. Allergic rhinitis is a chronic upper respiratory disease, with inflammation in the nasal mucosa mediated by immunoglobulin E (IgE) [4, 5]. House dust mite (HDM) is the characteristic allergic trigger for perennial AR, which occurs throughout the year [2]. The main species of mite is *Dermatophagoides pteronyssinus*, and Der p1 is its most allergenic protein with protease activity [6]. Exposure of hypersensitive individuals to allergens elicits two types of allergic responses – the early phase and the late phase. The early allergic reaction is characterized by sneezing, an itchy nose, and rhinorrhea. The late allergic response is characterized by an increased number of eosinophils, which causes nasal congestion, chronic wheeze, and inflammation [2, 7].

Over the past decade, several research groups reported that allergen immunotherapy is a promising allergic healing involving long-term induced remission and prevention of new sensitization of patients with a definite history of sensitization [8, 9]. In this context, a product with HDM allergenic extract has been developed using Indonesian HDM (IHDM) to diagnose the sensitivity of patients to HDM allergen. The HDM allergenic extracts originating from different regions and prepared by different manufacturers are expected to have different allergenicity [10–12] and have been reported to induce a variety of T-cell responses and IgE binding due to different sequences of the allergenic protein, diversity of raw materials, and different compositions [13–15].

This study aimed to optimize the allergenic rhinitis mouse model that was generated using IHDM for triggering AR sensitivity to test the *in vivo* sensitivity and safety of this product. Materials and Methods

### Ethical approval

All protocols in this study complied with the Guidelines for the Care and Use of Laboratory Animals issued by the National Institutes of Health revised in 1985 and were approved by the Research Ethics Commission of the Faculty of Veterinary Medicine, Universitas Airlangga (Number: 2.KE.058.05.2021).

### Study period and location

The study was conducted from March to December 2021 at the Animal Laboratory, *In Vitro* Laboratory (Biomolecular) Research Center, Faculty of Pharmacy, Universitas Airlangga, and Pathology-Anatomy Laboratory of the Faculty of Medicine, Universitas Airlangga.

### Animals

Healthy nulliparous non-pregnant female BALB/c mice, aged 6–8 weeks and weighing 20–25 g, were used for the sensitivity test. Sex selection of experimental animals needs to be based on the goals and limitation that exists in a particular sex [16, 17]. In AR conditions, several studies [18–20] have shown a higher sensitivity in showing allergic responses in female mice compared to males, as evidenced by the higher concentrations of pro-inflammatory cytokines produced. This is the main reason for using female sex to see the molecular responses more clearly. Mice were adapted for 1 week in ventilated cages, with a maximum of six animals per cage, under 12 h light/12 h dark cycle at controlled ambient temperature (23 ± 2°C) with *ad libitum* access to drinking water and standard pelleted laboratory diet during the course of experiment [21, 22].

### Allergenic extract

The IHDM extract (5 mg/mL), used as the allergen, was provided by Dr. Soetomo Regional Hospital (Surabaya, Indonesia) and was prepared with dust originating from Indonesia containing 11.3–26.6 ng/mL Der p1. The extract was prepared in sterile normal saline (NaCl 0.9%) and was administered through intraperitoneal and intranasal routes.

### Animals and grouping

Fifty-six mice were used for the sensitivity test. The animals were divided into seven groups of eight mice each, as follows: (1) Normal group, with NaCl sensitization and challenge; (2) negative control group, with NaCl sensitization and IHDM challenge; (3) positive control group, with NaCl sensitization and 0.1% histamine challenge; and four groups of AR caused by IHDM-induced sensitization and challenged with IHDM, namely, (4) low-dose group, with 12.5 μg IHDM; (5) moderate-dose group, with 50 μg IHDM; (6) high-dose group, with 250 μg IHDM; and (7) very high-dose group, with 500 μg IHDM. Every IHDM sensitization was given together with 2 mg aluminum hydroxide (alum), as an allergic adjuvant, and every IHDM challenge was with 31.25 μg IHDM extract only.

### Sensitization protocol and allergen exposure

**Figure-1:**
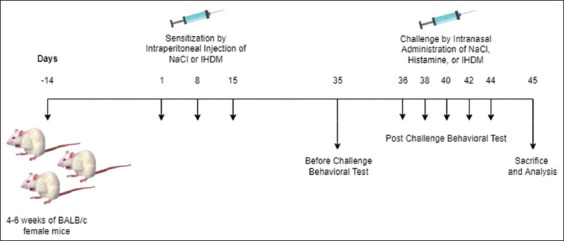
Experimental treatment protocol.

The AR sensitivity was triggered by exposing mice to 200 μL diluted IHDM-alum on days 1, 8, and 15 through intraperitoneal injection (Groups 4–7). Mice were sensitized with 200 μL NaCl intraperitoneally on days 1, 8, and 15 (Groups 1–3). Three weeks after the last sensitization, the challenge was given intranasally 5 times on alternate days – 36, 38, 40, 42, and 44. The IHDM challenge was given intranasally with 25 μL diluted IHDM extract (Groups 2 and 4–7), NaCl (25 μL) challenge was given intranasally (Group 1), and 0.1% histamine (25 μL) challenge was given intranasally (Group 3). Each dilution was done using 0.9% NaCl. Mice were sacrificed on day 45 (Figure-1).

### Evaluation of symptoms

Two observers, who were blinded to this experiment, counted the frequency of nose rubbing and sneezing events during a 15 min period after the last allergen challenge using the video recording of the mice [15, 23]. The evaluations were carried out 6 times during this experiment, once a day before the challenge (before the challenge behavior test) and 5 times each after the challenge had been given (post-challenge behavior test).

### Assessment of the expression of interleukin-5 (IL-5) mRNA in the nasal mucosa using semi-quantitative reverse transcription-polymerase chain reaction (RT-PCR)

The nasal mucosa was removed after the mice were sacrificed. Total RNA was extracted from the nasal mucosa cell using the Total RNA Purification Kit (Jena Bioscience, Germany) and quantitated using the QuantiFluor^®^ RNA Sample Kit (Promega, USA). Next, first-strand cDNA was synthesized using the GoScript™ Reverse Transcription System (Promega). DNA amplification was performed using the GoTaq Green Master Mix (Promega). Both cDNA synthesis and DNA amplification were performed on a thermal cycler (Applied Biosystems, USA). The reaction for cDNA synthesis was carried out at 25°C for 5 min and 42°C for 60 min. While, for DNA amplification, the pre-denaturation was performed at 94°C for 5 min and the reaction was carried out at 94°C for 40 s, 55°C for 60 s, and 72°C for 7 min. The reaction was repeated for 40 cycles. The oligonucleotide primer sequences used were as follows: IL-5 forward primer, 5′-ATGGAGATTCCCATGAGCAC-3′ and reverse primer, 5′-GTCTCTCCTCGCCACACTTC-3′; GAPDH forward primer, 5′-CGGAGTCA ACGGATTTGGTCGTAT-3′ and reverse primer, 5′-AGCCTTCTCCATGGTGGTGAAGAC-3′. The mRNA levels of IL-5 and GAPDH were determined using semi-quantitative RT-PCR, and the amplified products were visualized after electrophoresis on a 2% agarose gel (Invitrogen, USA). The intensity of the amplified bands was measured using the ImageJ software (NIH, Bethesda, MD, USA) [24].

### Histopathological analysis

At 24 h after the last challenge, mice were decapitated, and the head was fixed in 10% neutral buffered formalin at room temperature (25 ± 2°C) for 7 days. The head tissues were then embedded in paraffin, and 4 mm coronal sections were stained with hematoxylin and eosin (H and E). The number of eosinophils in the nasal mucosa was counted under a light microscope (with ×400; Olympus TH4-200, Japan). The data show the mean of the results from five random areas for each mouse nasal mucosa sample [25].

### Statistical analysis

Data show the mean ± standard error of the mean. Symptom scores (number of nose rubbing and sneezing) obtained were analyzed statistically using a two-way analysis of variance (ANOVA) followed by Tukey’s *post hoc* test. The data for the expression of IL-5 mRNA and cell counts for eosinophil infiltrations were analyzed statistically using one-way ANOVA followed by Tukey’s *post hoc* test. p < 0.05 was considered statistically significant. All statistical analyses were performed using GraphPad Prism version 9.0.2 software (GraphPad Software Inc., California, USA).

## Results

### Sensitization with the IHDM allergenic extract increased the number of nose rubbing

**Figure-2:**
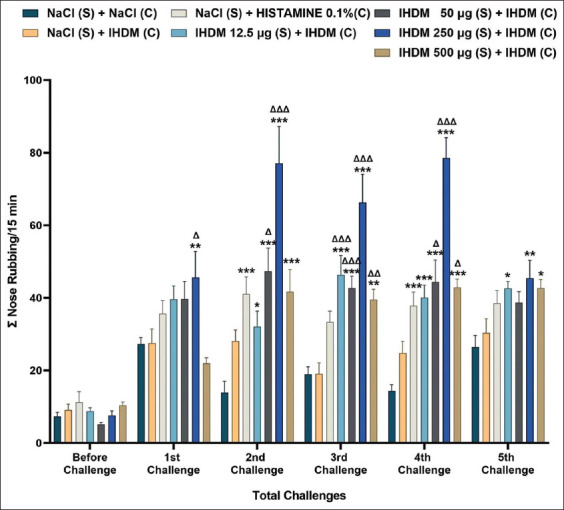
Indonesian house dust mite (IHDM) allergenic extract sensitization increased the number of nose rubbing in allergenic rhinitis (AR) mice models. Data are shown as mean ± standard error of the mean (n = 8) for each treatment group: Normal group (NaCl sensitization and challenge); negative control group (NaCl sensitization, IHDM challenge); positive control group (NaCl sensitization, 0.1% histamine challenge); low-dose group (12.5 µg IHDM sensitization, IHDM challenge); moderate-dose group (50 µg IHDM sensitization, IHDM challenge); high-dose group (250 µg IHDM sensitization, IHDM challenge); and very high-dose group (500 µg IHDM sensitization, IHDM challenge). For the treatment in each group: (S): Sensitization; (C): Challenge. ***p < 0.001, **p < 0.01, *p < 0.05 versus the normal group. ΔΔΔp < 0.001, ΔΔp < 0.01, Δp < 0.05 versus the negative control group.

The induction of AR with IHDM sensitization significantly increased the number of nose rubbing compared with that in normal and negative control groups (Figure-2). The increase happened after the 2^nd^ challenge in the low- and very high-dose groups compared with that in the normal group, and after the 3^rd^ challenge when compared with that in the negative control group. In the moderate-dose group, the increase in the number of nose rubbing occurred after the 2^nd^ challenge compared with that in the normal and negative control groups. The rubbing increased after the 1^st^ challenge in the high-dose group compared with that in the normal and negative control groups. The 0.1% histamine challenge in the positive control group also significantly increased the number of nose rubbing compared with that in the normal group after the 2^nd^ and 4^th^ challenges. However, only IHDM challenge in the negative control group did not increase the nose rubbing behavior compared with that in the normal group. These results show that the treatments and the number of challenges, as well as the interaction between these two factors, could affect the number of nose rubbing (two-way ANOVA, p < 0.001).

### Sensitization with the IHDM allergenic extract increased the number of sneezing

**Figure-3:**
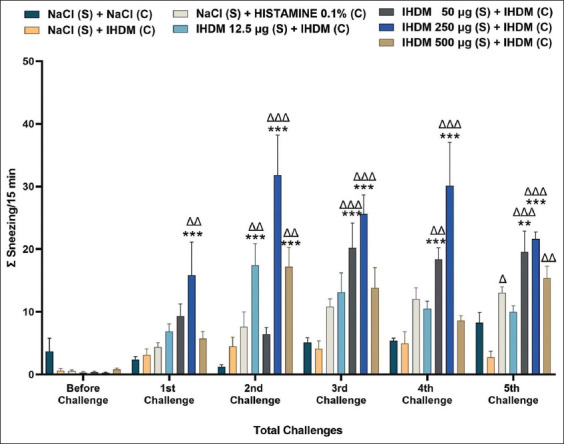
Indonesian house dust mite (IHDM) allergenic extract sensitization increased the number of sneezing in allergenic rhinitis (AR) mice models. Data are shown as mean ± standard error of the mean (n = 8) for each treatment group: Normal group (NaCl sensitization and challenge); negative control group (NaCl sensitization, IHDM challenge); positive control group (NaCl sensitization, 0.1% histamine challenge); low-dose group (12.5 µg IHDM sensitization, IHDM challenge); moderate-dose group (50 µg IHDM sensitization, IHDM challenge); high-dose group (250 µg IHDM sensitization, IHDM challenge); and very high-dose group (500 µg IHDM sensitization, IHDM challenge). For the treatment in each group: (S): Sensitization; (C): Challenge. ***p < 0.001, **p < 0.01 versus the normal group. ΔΔΔp < 0.001, ΔΔp < 0.01, Δp < 0.05 versus the negative control group.

The induction of AR with IHDM sensitization significantly increased the number of sneezing compared with that in the normal and negative control groups (Figure-3). The increase happened after the 2^nd^ challenge for the low-dose group compared with that in normal and negative control groups. In the moderate-dose group, an increase was observed after the 3^rd^ challenge compared with that in the normal and negative control groups. In the very high-dose group, the increase in sneezing occurred after the 2^nd^ challenge compared with that in the normal group and after the 2^nd^ and 5^th^ challenges compared with that in the negative control group. In the high-dose group, sneezing increased after the 1^st^ challenge compared with that in the normal and negative control groups. The 0.1% histamine challenge in the positive control group also significantly increased the number of sneezing compared with that in the negative control group after the 5^th^ challenge. As for the nose rubbing behavior, only the IHDM challenge in the negative control group did not increase the sneezing behavior compared with that in the normal group. These results also show that the treatments and the number of challenges, as well as the interaction between these factors, could affect the number of sneezing (two-way ANOVA, p < 0.001).

### Effect of IHDM allergenic extract on the expression of IL-5 mRNA in the nasal mucosa

**Figure-4:**
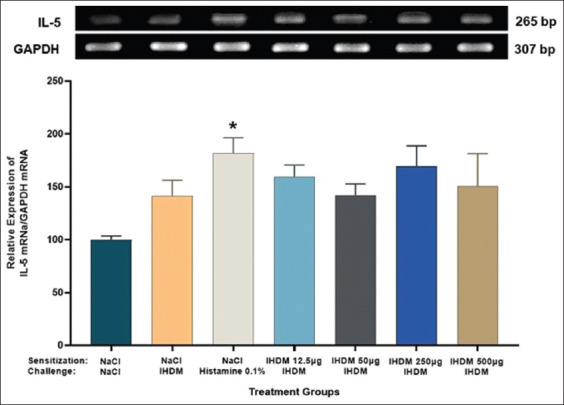
Indonesian house dust mite (IHDM) allergenic extract sensitization effects on the expression of interleukin-5 (IL-5) mRNA in mouse nasal mucosa tissue. There were no significant changes; however, a trend of increase in the expression of IL-5 mRNA was observed. Data are shown as mean ± standard error of the mean (n = 4) for each treatment group: Normal group (NaCl sensitization and challenge); negative control group (NaCl sensitization, IHDM challenge); positive control group (NaCl sensitization, 0.1% histamine challenge); low-dose group (12.5 µg IHDM sensitization, IHDM challenge); moderate-dose group (50 µg IHDM sensitization, IHDM challenge); high-dose group (250 µg IHDM sensitization, IHDM challenge); and very high-dose group (500 µg IHDM sensitization, IHDM challenge). For the treatment in each group: (S): Sensitization; (C): Challenge. *p < 0.05 versus the normal group.

No significant changes in the expression of IL-5 mRNA were observed in the mouse nasal mucosa from the AR groups with IHDM-induced sensitization (Figure-4). On the contrary, a tendency of increased expression of IL-5 mRNA was observed. Only the IHDM challenge also did not increase the expression of IL-5 mRNA in the nasal mucosa compared with that in the negative control group. However, the 0.1% histamine challenge induced an increase in the expression of IL-5 mRNA compared with that in the normal group (p < 0.05).

### Sensitization with the IHDM allergenic extract increased eosinophil infiltration in the nasal mucosa

**Figure-5:**
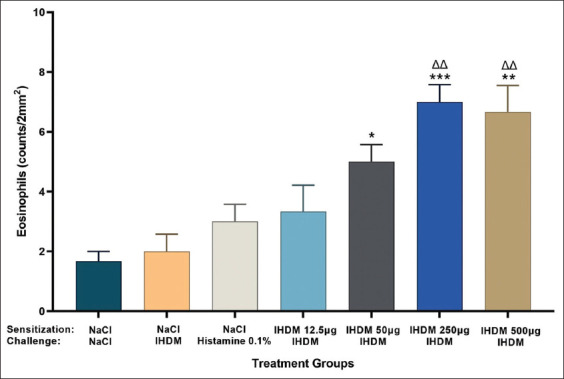
Indonesian house dust mite (IHDM) allergenic extract sensitization increased infiltration of eosinophils in the mouse nasal mucosa tissue. Data are shown as mean ± standard error of the mean (n = 3) for each treatment group: normal group (NaCl sensitization and challenge); negative control group (NaCl sensitization, IHDM challenge); positive control group (NaCl sensitization, 0.1% histamine challenge); low-dose group (12.5 µg IHDM sensitization, IHDM challenge); moderate-dose group (50 µg IHDM sensitization, IHDM challenge); high-dose group (250 µg IHDM sensitization, IHDM challenge); and very high-dose group (500 µg IHDM sensitization, IHDM challenge). For the treatment in each group: (S): Sensitization; (C): Challenge. ***p < 0.001, **p < 0.01, *p < 0.05 versus the normal group. ΔΔp < 0.01 versus the negative control group.

**Figure-6:**
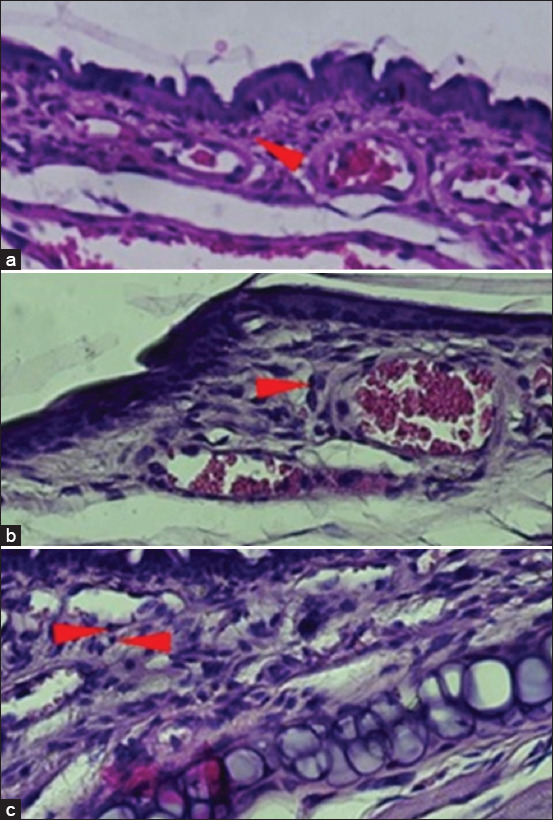
Hematoxylin and eosin staining (×400) of the nasal mucosa tissue from experimental mice (I). The groups based on the treatment were as follows: Normal group with NaCl sensitization and challenge (a); negative control group with NaCl sensitization and Indonesian house dust mite challenge (b); and positive control group with NaCl sensitization and 0.1% histamine challenge (c). Red arrowheads indicate eosinophils.

**Figure-7:**
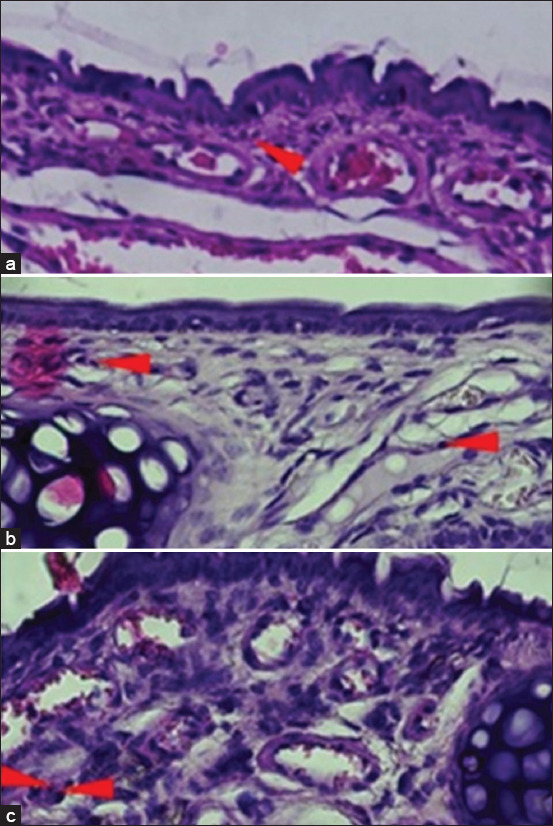
Hematoxylin and eosin staining (×400) of the nasal mucosa tissue from experimental mice (II). The groups based on the treatment were as follows: Normal group with NaCl sensitization and challenge (a); low-dose group with 12.5 µg Indonesian house dust mite (IHDM) sensitization and IHDM challenge (b); and moderate-dose group with 50 µg IHDM sensitization and IHDM challenge (c). Red arrowheads indicate eosinophils.

**Figure-8:**
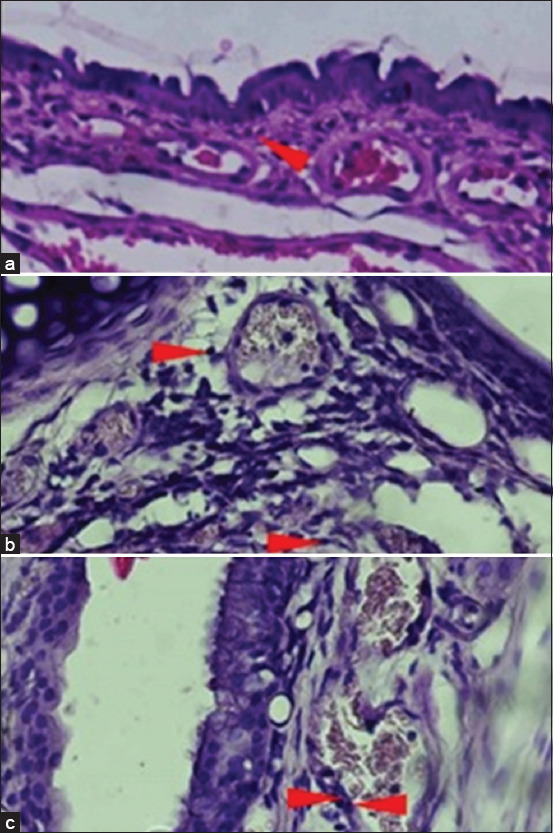
Hematoxylin and eosin staining (×400) of the nasal mucosa tissue from experimental mice (III). The groups based on the treatment were as follows: Normal group with NaCl sensitization and challenge (a); high-dose group with 250 µg Indonesian house dust mite (IHDM) sensitization and IHDM challenge (b); and very high-dose group with 500 µg IHDM sensitization and IHDM challenge (c). Red arrowheads indicate eosinophils.

The induction of AR with IHDM sensitization significantly increased the infiltration of eosinophils in the nasal mucosa (Figure-5). The H and E staining of coronal sections of the nasal tissue from the AR model groups also revealed increased eosinophil infiltration compared with that in normal and negative control groups (Figures-6–8). The increased eosinophil infiltration occurred in the high- and very high-dose groups compared with that in normal and negative control groups. Moreover, the increase in eosinophil infiltration was dose-dependent and was observed after the administration of the three initial doses of the IHDM allergenic extract. There was no significant change in eosinophil infiltration in both the negative and positive control groups. However, a tendency of increase in eosinophil infiltration was observed in the positive control group compared with that in the normal group.

## Discussion

This study was conducted to ensure the effectiveness of the IHDM allergenic extract produced originally in Indonesia. House dust mite is one of the persistent indoor allergens. Exposure of hypersensitive individuals to house dust mite allergenic protein through the nose can trigger allergic responses, such as AR [1, 2, 26]. HDM allergenic extracts have been prepared in some countries to help physicians create a precise diagnosis of a patient’s allergy [9]. The effectiveness of an allergenic extract is generally judged by determining how it triggers a specific allergic response. For example, in AR, the hallmark allergic response is an itchy nose, sneezing, and the involvement of inflammatory cells in the nasal mucosa [1, 27].

Based on the evaluation of symptoms, the number of nose rubbing and sneezing showed that the IHDM sensitizing in AR mice model groups triggers AR sensitivity. These findings indicated the mice in the four groups with IHDM sensitization experienced the early phase AR response. The use of AR mice models for this kind of behavior validation test has been reported previously [23, 28, 29]. The present study is, however, the first behavior test report using an AR mice model showing an interaction between the number of challenges and the different treatments in affecting the nose rubbing and sneezing behavior.

The expression of IL-5 mRNA in the nasal mucosa was not increased in IHDM-induced AR groups, but only a tendency to increase was observed, which could be a sign of the onset of the late phase allergic response in AR. These results are in agreement with those reported by Lee *et al*. [28], who showed different expression levels of IL-5 mRNA in the nasal mucosa and mice splenocyte cultures. The absence of a significant change in IL-5 expression in the present study is presumably due to various chemotactic factors that caused a low expression of IL-5 in the nasal mucosa. Another cause could be the duration of the IL-5 mRNA translation to IL-5 cytokine; it is possible that the amount of protein was more than that of the mRNA at the time of processing the samples. This could result in low levels of detectable IL-5 mRNAs [30–32]. The trend of increased IL-5 mRNA expression compared with that in the normal group shows an important role of IL-5 in the pathogenesis of AR [1, 25, 33].

The results of eosinophil infiltration indicate the involvement of inflammatory cells, mainly eosinophils, in nasal inflammation, as a late-phase allergic response in AR [1, 7]. Although a significant increase was not observed in the low-dose group, the eosinophil infiltration response to IHDM sensitization was found to depend on the initial dose of IHDM used for sensitization. The very high dose resulted in a similar result as that obtained with the high dose. Thus, the infiltration of eosinophils into the nasal mucosa resulted from an allergic reaction. Such an increase in eosinophil infiltration in the nasal mucosa has been reported previously [23, 25, 28, 33].

The study show that IHDM sensitization is essential and effective in triggering AR sensitivity in the mouse model. The negative control group indicated its importance and effectiveness without IHDM sensitization. However, with the IHDM challenge, there were no significant differences in AR sensitivity compared with that in the normal group, probably because the mice did not experience the sensitization phase [7].

Unlike the mice in the negative control group, those in the positive control group did not experience the sensitization phase (sensitization with NaCl and 0.1% histamine challenge). Nonetheless, mice in this group experienced clinical symptoms and molecular mechanisms of AR. The activity of histamine caused this after the challenge which binds to its receptors, such as H_1_, H_2_, and H_4_. The activation of the H_1_ receptor on sensory nerve endings can result in sneezing and pruritus. The activation of H_1_ and H_2_ receptors on mucosal blood vessels leads to nasal congestion and plasma leakage [34]. The activation of the H_4_ receptor on dendritic cells induces Th2 responses against allergen [35]. However, there was no significant increase in eosinophil infiltration in this group. This is due to the effect of histamine on eosinophil migration depending on the dose given. Therefore, the results of eosinophil infiltration in this study obtained at high doses might be due to the inhibition of eosinophil chemotaxis through the involvement of the H_2_ receptor [36]_._

We also noted a paradoxical event with regard to the symptoms in the high-dose and very high-dose groups. Lower nose rubbing and sneezing were observed in the very high-dose group compared with that in the high-dose group. This behavioral change is thought to be caused by the administration of a very high dose of the allergenic extract, which could have modified the natural mechanism of RA pathogenesis as a form of immune tolerance to antigens [1, 28, 37]. These findings indicate that IHDM allergic extract has great potential as an effective immunotherapy agent for allergies. However, further research is needed in this regard.

Another finding in this study is the non-linearity of the results of the behavioral evaluation in the animal models between the high- and very high-dose groups. Similar was the case when comparing molecular and histopathological results. This could be due to the following reasons: (1) IL-5 mRNA is produced by Th2, mast, and ILC2 cells. Therefore, the exact profile of IL-5 mRNA levels would depend on the expression in these cells [38]; (2) the increase in IL-5 levels could also affect survival and lead to the prevention of eosinophil apoptosis; therefore, the number of eosinophil cells in the nasal mucosa was high. In addition, eosinophil infiltration is also induced by eotaxin-1/CCL11 as an innate immune response [39]; (3) the different biomolecular pathways underlying the clinical symptoms associated with eosinophil infiltration involve IL-4 and IL-13, and the major cytokine is IL-5. Therefore, there could be a difference in the increase in IL-4 and IL-13 levels with that in IL-5 levels, considering the paradoxical effect that was observed [7, 40]. However, this phenomenon still needs to be explored to explain the mechanism with regard to the expression of IL-4 and IL-13 mRNA. The nasal hyper-responsiveness is also thought to be due to eosinophil infiltration [1, 28].

## Conclusion

The data obtained in this study indicate that the IHDM allergenic extract could be an effective sensitizing agent in triggering AR sensitivity in mice models. However, further research is needed to study the effect of IHDM sensitization on paradoxical properties and the *in vivo* effectiveness of IHDM allergen extract as an immunotherapy agent for treating allergies. These results will pave the way for devising a strategy for allergy therapy. The limitation of this study is in the use of the IHDM, which is expected to be different from an extract prepared from other sources of HDM. This study should provide a proper model for preclinical studies on *in vivo* testing of the effectiveness of immunotherapy against AR.

## Authors’ Contributions

JK: Conceptualization, methodology, supervision, and manuscript reviewing. CA and ADN: Validation, formal analysis, and resources management. YAP, FD, and WFS: Data collection and manuscript drafting. All authors have read and approved the final manuscript.
